# Algorithm for appearance simulation of plant diseases based on symptom classification

**DOI:** 10.3389/fpls.2022.935157

**Published:** 2022-07-18

**Authors:** Meng Yang, Shu Ding

**Affiliations:** ^1^School of Information Science and Technology, Beijing Forestry University, Beijing, China; ^2^Engineering Research Center for Forestry-Oriented Intelligent Information Processing of National Forestry and Grassland Administration, Beijing, China

**Keywords:** plant disease simulation, symptom classification, time-varying model, deep learning, similarity test

## Abstract

Plant disease visualization simulation belongs to an important research area at the intersection of computer application technology and plant pathology. However, due to the variety of plant diseases and their complex causes, how to achieve realistic, flexible, and universal plant disease simulation is still a problem to be explored in depth. Based on the principles of plant disease prediction, a time-varying generic model of diseases affected by common environmental factors was established, and interactive environmental parameters such as temperature, humidity, and time were set to express the plant disease spread and color change processes through a unified calculation. Using the apparent symptoms as the basis for plant disease classification, simulation algorithms for different symptom types were propose. The composition of disease spots was deconstructed from a computer simulation perspective, and the simulation of plant diseases with symptoms such as discoloration, powdery mildew, ring pattern, rust spot, and scatter was realized based on the combined application of visualization techniques such as image processing, noise optimization and texture synthesis. To verify the effectiveness of the algorithm, a simulation similarity test method based on deep learning was proposed to test the similarity with the recognition accuracy of symptom types, and the overall accuracy reaches 87%. The experimental results showed that the algorithm in this paper can realistically and effectively simulate five common plant disease forms. It provided a useful reference for the popularization of plant disease knowledge and visualization teaching, and also had certain research value and application value in the fields of film and television advertising, games, and entertainment.

## Introduction

Dynamic visualization of plant diseases (Dickinson, 2020) can not only promote the development of agricultural informatics, but also has important implications for the study of plant phenomics (Pieruschka and Schurr, 2019). At a time of recurring epidemics, it can provide an innovative approach to the traditional study of plant diseases and can add interest to teaching in agriculture and forestry. It also exists in increasing demand in the film and television advertising and game entertainment industries, and can be applied to virtual space construction, virtual reality (VR) interaction, and game specific scene modeling. Combining the interrelationship between disease and environment in plant disease ecology and the description of plant disease pathogenesis patterns in epidemiology, one of the hot issues today is the realization of reasonable and realistic plant disease simulations.

Plant disease visualization simulation includes the simulation of characteristics such as disease spot distribution, color, geometry and textural properties. Kider et al. (2011) developed a fungal-bacterial reaction-diffusion model to parameterize the physical properties involved in fruit decay as a way to simulate the aging and decay process of fruits. Based on this, Fan et al. (2013) used an improved reaction-diffusion model to model the appearance of fruit ring-spot decay. Miao et al. (2014) modeled the spatial movement of cucumber powdery mildew spots using the cellular texture proposed by Worley (1996) to model the mildew layer formed by powdery mildew using Shell rendering, taking the distribution, movement mode, and final morphology of the spots as three spatial information of the spots. Xu et al. (2017) proposed a time-varying appearance model by extracting information on the apparent characteristics of the disease from real disease images and reasonably extrapolating the disease spot infestation process, which was applied to the apparent modeling of plant diseases. Liu and Fan (2015) proposed a modified plasto-spring model combined with cell mechanics to implement simulation modeling of fruit sunburn disease. Wu et al. (2018) proposed a 3D visualization model for controlling the fruit decay process using global decay parameters and decay resistance parameters, which can flexibly and quickly perform each point on the fruit model manipulated to complete the simulation of fruit shape deformation and decay appearance. Leaf discoloration or wilting is also a manifestation symptom of common plant diseases. Tang et al. (2013) implemented leaf deformation based on a modified mass-spring model, which regarded the color change as a sequence of continuous discrete states, and combined these two parts based on a Markov chain model to realize the leaf change process under different environmental parameter settings. Jeong et al. (2013) represented the leaf as a triangular-Voronoi bilayer structure and simulated the complex curl and fold of the leaf by uneven contraction. As can be seen from the above, there are abundant studies related to plant disease simulation, but most of the proposed simulation algorithms are aimed at a particular plant disease symptom to analyze its apparent morphological characteristics to realize the simulation, lacking the exploration of common problems existing in different plant diseases, with complicated methods and large constraints.

Plant morphology can reflect the gene expression, reproductive growth and resource acquisition of plants. The implementation of morphological modeling of plants using computer languages, as opposed to the graphical information of plants kept in the form of pictures, is also an important reference for this paper. Geometric topology-based modeling is the closest modeling approach to plant morphological structure. Chen et al. (2018) extracted modeling constraint rules and improved the parametric L system to generate complex 3D models of trees based on tree observation data and forestry theory knowledge. Wen et al. (2021) defined the mathematical representation of 3D plant nodes, specified the conversion method between its skeleton model and network model, and completed the plant population of different maize varieties by assembling 3D plant nodes 3D modeling. Such methods generate models with a strong sense of realism, but require professionals to provide specific plant growth rules and parameters that can describe plant morphology, which is more difficult for non-agroforestry professional users. Sketch-based modeling is a relatively flexible and interactive approach. Liu et al. (2019) built a system for interactive modeling of trees in VR based on 3D gestures with the help of a head-mounted display and a 6-DOF motion controller for interaction. Zhang et al. (2021) defined 3D sketches drawn by users in VR as an envelope of tree leaves and trunks that can automatically generate a complete 3D-tree model, and it can be edited twice. Such methods support direct user control over the generation of plant forms, but there are trade-offs to be made in terms of interactivity, usability, and fine-grained control over plant forms. Modeling based on measurement data mainly includes image-based and point cloud-based modeling. Chen et al. (2017) proposed a hierarchical denoising method based on multi-viewpoint image sequences to build 3D models of crops in order to improve the accuracy of 3D point cloud reconstruction. Liu et al. (2021) used conditional generative adversarial networks to predict the 3D skeleton of trees from individual images and 2D contours drawn by users, respectively. A tree model was generated using procedural modeling techniques. Such methods often face problems such as expensive collection equipment and cumbersome data processing. Curvilinear surface-based plant morphology modeling can better establish the connection between morphological structure and physiological function. Alsweis et al. (2017) extracted image contours using a curvature-scale spatial angle detection algorithm and proposed a procedural biologically motivated method to model leaf vein morphology at different levels. Isokane et al. (2018) used Bayesian expansion to infer plant branching probabilities and proposed a method to observe and infer the 3D plant branching structure hidden beneath the leaves from multiple perspectives. Such methods need to ensure smooth and continuous boundary, complicated operation and low efficiency of the algorithm. In general, the above approaches to modeling plant morphology have mainly focused on the organ structure and growth changes of the plant itself in a healthy state, while modeling the morphology of plants affected by disease infestation is lacking.

The phenomena of discoloration, aging, and corrosion occurring on the surface of an object are to some extent common to the different disease symptoms on the plant epidermis due to disease infestation, so the research on texture simulation can provide effective reference for the simulation of plant diseases. Zhang et al. (2014) extracted the texture features of real rust spot pictures, which can be selected and set texture weights when drawing the model parameters to achieve texture blending and obtain different states of rust simulation. Kamata et al. (2014) considered the factors of surface geometric features (convexity, occlusion, orientation, and location) of metals and their anticorrosive coating peeling areas for corrosion calculations to simulate corrosion phenomena in peeling areas. Bellini et al. (2016) calculated the estimated age map of weathering phenomena in a texture of a given input image based on the prevalence of plaque-like patches in that image, generated a complete weathering texture and simulate the de-weathering and weathering processes. Zhang et al. (2018) proposed a first-order quasi-static cracking node method (CNM) to simulate cracking in a 3D surface model and established a new stress and energy combined cracking criterion to deal with crack generation and extension. Munoz-Pandiella et al. (2018) proposed a technique based on a fast physics-inspired method that Ishitobi et al. (2020) used a triangular grid to simulate the weathering of a rust-proof coated metal surface after mechanical deterioration in three steps based on fundamental mechanics: “separation-splitting-exfoliation.” In texture representation and synthesis, Guingo et al. (2017) propose a two-layer representation of textures, with a noise layer capturing fine Gaussian patterns and a structure layer capturing non-Gaussian patterns and structures, synchronizing the two layers by a set of masks to make them consistent. Cavalier et al. (2019) propose a method based on local control of speckle noise by controlling the pulse distribution and a spatially defined kernel to create the desired texture appearance in a user-interactive manner. Due to the essential difference between the object of application and the principle of texture generation, a generation algorithm suitable for plant disease apparent texture needs to be explored on the basis of the reference.

In summary, it is of high research and application value to realize a plant disease visualization simulation with high realism, high universality and stable operation. In this paper, we deconstruct and analyze five common and distinctive disease symptom patterns in plant diseases, and propose a time-varying generic model of plant disease without violating the theory of plant pathology to show the dynamic process of plant disease infestation under different environmental conditions. Using disease symptoms as the basis for plant disease classification, we propose simulation algorithms corresponding to five common disease symptoms respectively, and realize visual simulation of different types of multiple plant diseases. Deep learning is used to check the similarity of simulation results in terms of the accuracy of symptom type recognition.

## Algorithms for plant disease simulation

### Time-varying generalized model for plant diseases

The infected host plant, the pathogenic agent and the environmental conditions conducive to disease development are known as “the disease triangle” (Scholthof, 2007). The occurrence of disease is the result of the fulfillment of these three necessary conditions. Therefore, the external environment in which the plant is located directly influences the growth of the plant and the spread of the disease. Meteorological factors are most closely related to the occurrence and prevalence of plant diseases, mainly including temperature, humidity, rainfall, light, wind, etc. In agroforestry research, data recording and analysis of environmental and plant diseases enable monitoring and prediction of plant disease development (Moyer et al., 2016; Shang et al., 2018; Chappell et al., 2020). The time-varying generic model is established with the principles of plant disease prediction as the main theoretical basis, and they are the three principles of continuity, analogy, and relevance. Due to professional and equipment limitations and lack of accurate meteorological measurement data, this paper combines the description of the process of different plant disease epidemics, ignores the influence of other factors, and assumes that the acceleration of the actual spread of disease spots is mainly influenced by two conditions: atmospheric temperature and humidity. Within the scope of the existence of unidirectional effects of temperature and humidity on phytophthora, a time-varying generic model is developed to represent the common relationship between different plant diseases under the influence of environmental conditions.

For any plant disease, define the current spot morphology as State, expressed as Equation (1):


(1) $$\mathrm{State}=\left[\begin{array}{c} M\\ C \end{array}\right]$$

where *M* refers to *M*_1_, *M*_2_, *M*_3_, *M*_4_, and *M*_5_ in sequence for the disease symptom types described in the text in the actual code operations, denoted as variable parameters controlling the extent (size or number) of disease spot spread in the simulation algorithm. It will be described in detail in specific sections. *C* denotes the color component matrix of the disease spots.

In this paper, *V* denotes the rate of disease diffusion, *a* denotes the acceleration of disease spot diffusion, and *t* denotes the diffusion time. To simplify the model and ignore the influence of other factors, the actual acceleration of disease spot diffusion is assumed to be influenced by two conditions: atmospheric temperature and humidity, and a quantitative relationship of uniform form is established in the range where temperature and humidity play a unidirectional role on plant diseases. Tan (1991) had proposed the Richards function as a general model to simulate the temporal dynamics of plant disease epidemics, and through detailed derivation, proved that it can reflect the epidemiological pattern of many plant diseases. Based on this theoretical formula, the following definition is made in this paper, as shown in Equations (2–4):


(2) M=Mmax∑i=1t(1-e-ViΔt)11-m      0≤m<1

(3) $$V_{i}=V_{i-1}+\left(\alpha \Delta T+\beta \Delta Q+\varepsilon \right)a\Delta t$$

(4) {ΔT=Ti-Ti-1ΔQ=Qi-Qi-1Δt=ti-ti-1      0<i<100

where *m* is the shape parameter of the growth curve, reflecting the type of disease growth function. α and β are the influence coefficients of temperature and humidity on the disease, respectively, and the corresponding values of α and β are different for different plant diseases. *T* denotes temperature (°C) and *Q* denotes relative atmospheric humidity (%). ε is the value of random error caused by other factors on acceleration, which is neglected in the actual operation. *M*_max_ is the maximum value of *M*.

For the color of the disease spot *C*, in order to make the color change process tend to be smooth, this paper uses the key frame linear interpolation method for simulation, and the color is updated once for each rendering of the screen. The color value in the most severe state is *C*_max_, and the initial spot color value is *C*_min_, then the color value *C* at time *t* is shown in Equation (5):


(5) $$C=C_{\min}+\frac{M}{M_{\max}}\cdot \frac{C_{\max}-C_{\min}}{t_{\max}}t$$

where *t*_max_ denotes the maximum value of the diffusion time.

### Types of symptoms with continuous area changes

#### Discoloration symptom simulation

Discoloration refers to a change in color of the diseased plant. In this section, Ginkgo yellows disease is selected for the study to simulate the discoloration symptoms.

**FIGURE 1:**
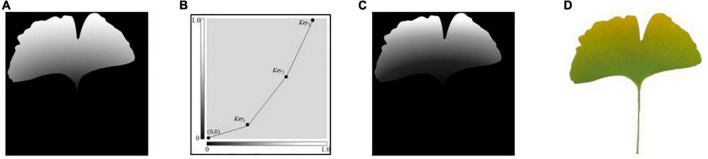
Schematic diagram of ginkgo yellows simulation. **(A)** Initial grayscale gradient map, **(B)** segmented grayscale conversion, **(C)** grayscale map after grayscale conversion, **(D)** rendering result.

The leaf yellowing shows a gradual process from green to yellow. As shown in Figure 1B, the grayscale remapping transformation is represented by a right-angle coordinate system, with the x-axis being the grayscale value before mapping and the y-axis being the grayscale value after mapping. In order to represent the color change process more richly, after normalization, the initial ginkgo grayscale gradient map (Figure 1A) is grayscale remapped. Three key points[*Key*_1_(*x*_1_, *y*_1_), *Key*_2_(*x*_2_, *y*_2_), *Key*_3_(*x*_3_, *y*_3_)] divide the whole process into three processing segments, and the default starting coordinates of the first segment are (0, 0), as shown in Equation (6):


(6) $$f_{g}\left(x\right)=\left\{ \begin{array}{c} \frac{y_{1}}{x_{1}}x,0\leq x\leq x_{1}\\ \frac{y_{2}-y_{1}}{x_{2}-x_{1}}\left(x-x_{1}\right)+y_{1},x_{1}\leq x\leq x_{2}\\ \frac{y_{3}-y_{2}}{x_{3}-x_{2}}\left(x-x_{2}\right)+y_{2},x_{2}\leq x\leq x_{3} \end{array}\right.$$

where *x*_1_, *y*_1_, *x*_2_, *y*_2_, *x*_3_, *y*_3_ are the exact values in practical application to determine the mapping function of each segment. Figure 1C shows the grayscale gradient after the three-stage mapping, where the coordinates of *Key*_1_, *Key*_2_, and *Key*_3_ are taken as (0.43, 0.14), (0.76, 0.60) and (0.94, 1.0), respectively, and the rendering results are shown in Figure 1D.

Keeping the vertical coordinates unchanged, the horizontal coordinates of *Key*_1_ and *Key*_2_ are dynamically assigned from large to small, and the amount of change is *M*_1_. The calculation of the grayscale mask image for generating uniformly discolored ginkgo yellowing disease is shown by Equation (7):


(7) $$I_{1}=f_{\text{g}}\left(I_{0}\left(x,y\right)\right)+Slope\cdot M_{1}$$

where (*x*, *y*) denotes the position of the pixel point. All image calculation formulas are performed simultaneously for each pixel point in the image, which essentially indicates the calculation of the value of each pixel point and is not repeated below. *I*_1_(*x*, *y*) denotes the image after segmented gray linear transformation, *I*_0_(*x*, *y*) denotes the initial gray gradient image, and *Slope* is the slope of the line between the key points, which takes the value of 1.4.

#### Powdery mildew symptom simulation

Powdery mildew symptom is characterized by the appearance of powdery or moldy material visible to the naked eye on the surface of the disease. In this paper, we take cucumber powdery mildew as the research object, use Worley noise to control the location of the occurrence of the disease spot and the geometry of the spot itself, and use Perlin noise (Perlin, 1985) to simulate the powdery mildew layer formed by the disease spot block to simulate the powdery mildew symptom.

**FIGURE 2:**
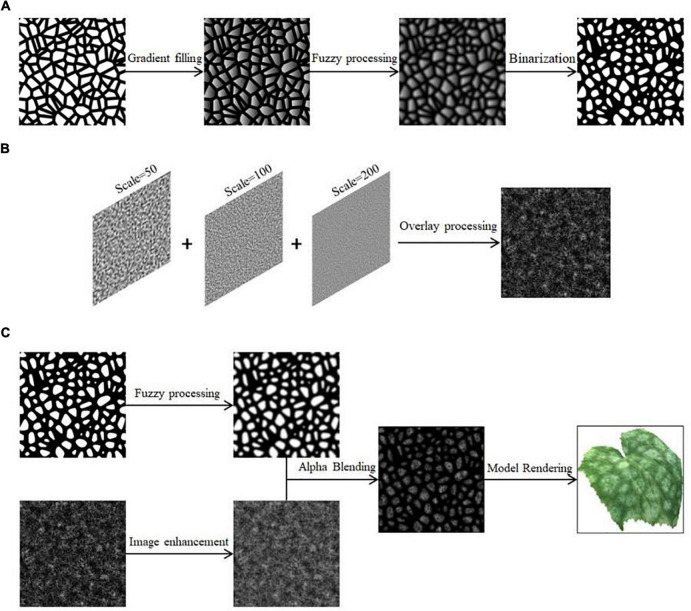
Schematic diagram of cucumber powdery mildew simulation. **(A)** Transformation of Worley noise, **(B)** Perlin noise superimposition, **(C)** generation of powdery mildew spot texture.

The texture edges of the Worley noise-like Voronoi map are clearly straight lines, so further transformations are needed. First, the Unity Shader is used to fill the noise after grayscale processing, so that the grayscale of each cell is randomized, and then blurred, and finally the threshold is set to binarize the image, so that the grayscale map can describe the shape of the lesion. After trial and error, a threshold value of 0.71 worked best. The process is shown in Figure 2A.

In order to reflect the granularity characteristics of the spots, the Perlin noise with different parameters is superimposed to generate fractal noise to simulate the effect of powdery mildew. It can be adjusted by changing the frequency and amplitude of the two parameters. Users can choose the number of superimpositions according to the actual simulation needs, and the generated Perlin noise function is shown in Equation (8):


(8) $$P\_Noise\left(x,y\right)=\sum_{i=0}^{n}\frac{Noise\left(2^{i}x,2^{i}y\right)}{Scale\left(i\right)}$$

where *Scale*() is the two-dimensional noise range, *n* is the number of noise functions superimposed, *Noise*() is the Perlin noise function. In this paper, we take *n* as 3, and the simulated noise effect after superposition is shown in Figure 2B.

Combining the above steps, Ahpha blending of the two in the Unity Shader generates a grayscale map of the spot texture of cucumber powdery mildew, which is *I*_2_(*x*, *y*), expressed by Equations (9, 10):


(9) $$I_{2}\left(x,y\right)=Alpha\cdot I_{\text{worley}}\left(x,y\right)+\left(1-Alpha\right)\cdot I_{\text{perlin}}\left(x,y\right)$$

(10) $$\left\{ \begin{array}{c} I_{\text{worley}}=\mathrm{Image}\left(W\_Noise\right)\\ I_{\mathrm{perlin}}=\mathrm{Image}\left(P\_Noise\right) \end{array}\right.$$

where *I*_worley_(*x*, *y*) is the grayscale image generated by Worley noise and *I*_perlin_(*x*, *y*) is the grayscale image generated by Perlin noise. After color mapping, the simulation result is rendered on the model, as shown in Figure 2C. The equal scale deflation of the crystals in Worley noise can control the size of the lesions. For some cells that are already small, the cells are scaled to a certain level and the small cells will disappear. Therefore, the cell is dynamically deflated from large to small to simulate the dynamic process of the spot from nothing to something, from small to large. The grayscale mapping representing the disease spots is updated in real time. The amount of deflation change is *M*_2_, as shown in Equation (11):


(11) $$W\_Noise={\mathrm{Noise}}_{\text{w}}\left(M_{2}\cdot Cell\right)$$

where *Noise*_w_() denotes the function that can deflate the cell size in noise and *Cell* denotes the cell in Worley noise.

### Types of symptoms of quantitative changes

#### Ring pattern symptom simulation

Ring pattern symptom is characterized by ring spot pattern. Initially, the plant surface produces brown, round, water-stained spots, which gradually form concentric whorls of varying shades of color as the spots spread. In this paper, we take apple ring rot as the specific object of study and describe the simulation of ring pattern symptom.

In this paper, the entire spot is split into two parts, the initial water-stained spot and the concentric whorl, and the generated gray-scale image of the spot morphology, which is *I*_3_(*x*, *y*), is expressed as Equation (12):


(12) $$I_{3}\left(x,y\right)=I_{\text{S}}\left(x,y\right)+I_{\text{Y}}\left(x,y\right)$$

where *I*_S_(*x*, *y*) is represented as a grayscale image of water-stained spots and *I*_Y_(*x*, *y*) is represented as a grayscale image of concentric whorls.

It is reasonably assumed that the small water-stained spots initially produced by the onset of disease determine the overall size, color basis, and outermost morphology of the spots as they spread and amplify. This part is disassembled step by step, and the regular circle is randomly perturbed by using Gaussian noise. Finally, the simulation results of this part are obtained by combining image operations, and the specific process steps are as follows.

**FIGURE 3:**
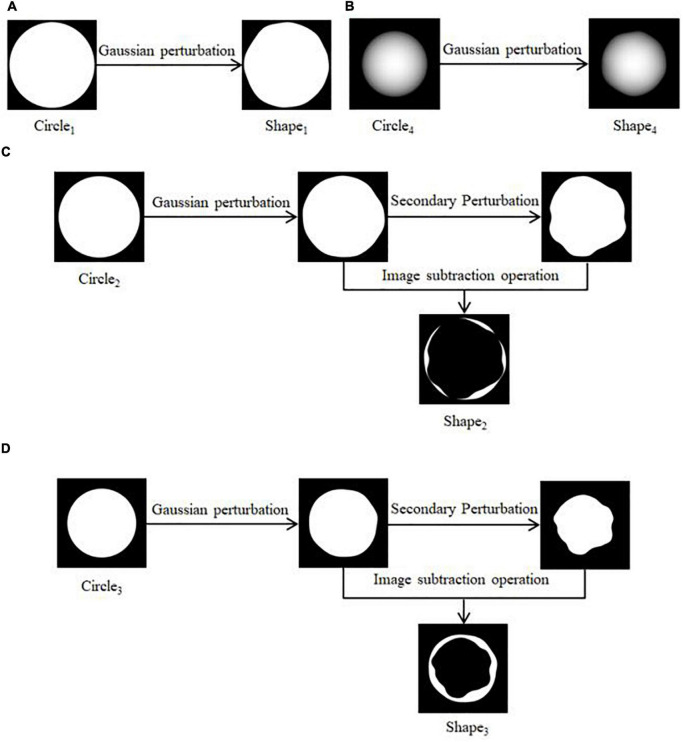
Pre-processing of shapes. **(A)** Generation of Shape_1_, **(B)** generation of Shape_4_, **(C)** generation of Shape_2_, **(D)** generation of Shape_3_.

**FIGURE 4:**
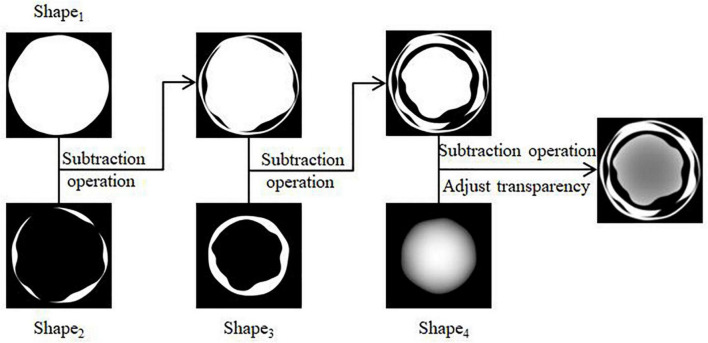
Generation of initial water stain-like spot.

(1) Four regular circular grayscale maps are generated in turn, with size satisfying Circle_1_ > Circle_2_ > Circle_3_ > Circle_4_. Shape_1_ and Shape_4_ are obtained by preprocessing Circle_1_ and Circle_4_. The result is shown in Figures 3A,B. Circle_2_ and Circle_3_ are perturbed by Gaussian twice, and the result after the first perturbation is subtracted from the result after the second perturbation to obtain Shape_2_ and Shape_3_. The result is shown in Figures 3C,D.
(2) The shape obtained in the above step is subtracted three times in turn, and the transparency of the result is adjusted to facilitate the subsequent superimposition of the whorl part to obtain the shape of the initial water-stained spots, as shown in Figure 4.

From a microscopic point of view, whorls are seen as formed by colonies in the process of continuous growth movement and cessation of aggregation. In this paper, each circle of the whorl from deep to shallow is regarded as a layer-by-layer radial gradient mapping that can be increased with time. The amount of change in the overall deflation of the spot shape is *M*_3_, and the current number of circles is determined by rounding the value of *M*_3_. Then the gray-scale image generation of the water-stained spot part is calculated as shown in Equations (13, 14):


(13) $$I_{\text{S}}\left(x,y\right)=\mathrm{Image}\left(Shape_{S}\right)$$

(14) $$Shape_{\text{S}}=M_{3}\cdot Shape$$

where *Shape* denotes the initial water-stained spot, *Shape*_S_ denotes the water-stained spot portion after deflation, and *Image*() denotes a function that converts the input into an image format of the same size as the plant texture mapping.

**FIGURE 5:**
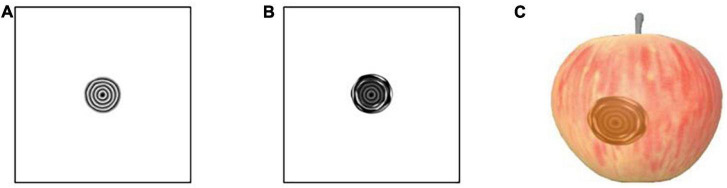
Schematic diagram of cucumber powdery mildew simulation. **(A)** Concentric circle map after random disturbance, **(B)** map after superposition of initial lesion, **(C)** rendering result.

The grayscale image generation for the concentric whorl section is calculated as shown in Equations (15, 16):


(15) $$I_{\text{Y}}\left(x,y\right)=\mathrm{Image}\left(Shape_{Y}\right)$$

(16) $$\begin{array}{c} Shape_{\text{Y}}=\mathrm{Rand}\left(\right)\cdot \sum_{i}^{N_{\text{turns}}}\mathrm{GradientMap}(\pi {\left(r_{0}+i\right)}^{2}\\ \;-\pi {\left(r_{0}+i-1\right)}^{2}) \end{array}$$

where *Rand*() is the random function for perturbation, *GradientMap*() is the gradient mapping function from 0 to 255, *r*_0_ is the radius of the initial circle, and *N*_Turns_ is the value of *M*_3_ rounded to represent the number of whorl circles. A random function is added to perturb the regular concentric whorl pattern (Figure 5A), which is closer to the real one. The new texture mapping map generated at each moment is continuously stored and updated. The mapping map of the initial water-stained spots after superimposed diffusion (Figure 5B) is rendered to obtain the results of apple whorl disease, as shown in Figure 5C.

#### Rust spot symptom simulation

Rust spot symptom is characterized by the appearance of different shaped spots on the plant surface formed by aggregations of small particles of varying sizes and distinctive projections. The rust spot symptom is simulated using wheat stripe rust as a specific study object.

**FIGURE 6:**
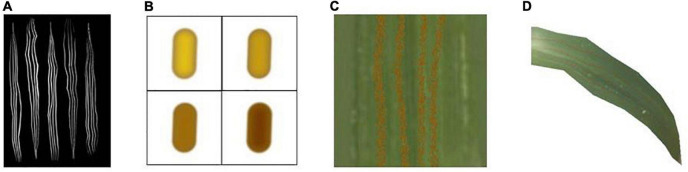
Schematic diagram of wheat stripe rust simulation. **(A)** Mask map, **(B)** particulate matter, **(C)** detail picture after bump treatment, **(D)** rendering result.

Based on the characteristics of wheat stripe rust in parallel strips, this paper uses mask mapping to mark the areas affected by wheat stripe rust. The white color is used to mark the areas where stripe rust develops, and the areas where it does not develop are marked in black. The corresponding mask map is shown in Figure 6A.

The generated spore mounds are viewed as consisting of a dense distribution of raised granules. In this paper, this granularity is represented by drawing a near-elliptical shape in two dimensions that can be used for gradient mapping, and the results after different color mapping are shown in Figure 6B. A number of granular points (the maximum number is 1,000*1,000) with 2 × 2 shape pixels are set, and the position distribution of the granules is randomly perturbed using a Gaussian random function.

In this paper, we use a normal map to simulate the bump of rust particles. When the type of normal texture is set to Normal map in Unity, the built-in function UnpackNormal can be used to properly sample the normal texture and extract the normal information by adjusting the bump level. The result is obtained by applying it to the Surface Shader for output. The detail is shown in Figure 6C. The rendering result is shown in Figure 6D.

In this paper, based on the description of the rust disease process, Unity Shader is used to update the mask mapping in real time based on dynamic color scale adjustment. This is able to simulate the dynamic process of disease spot from nothing to something and from sparse to dense in the actual rendering.

For grayscale images, the algorithm for input color scale adjustment is to first calculate the difference *Diff* between the white field threshold *threHigh* and the black field threshold *threShadow*. Then, the algorithm traverses each pixel in the mask mapping and calculates the difference *GrayDiff* between the input gray value *Gray* and *threShadow* for each pixel. If the value of *GrayDiff* is less than or equal to 0, the adjusted pixel gray value *Gray’* is 0. Otherwise, the adjusted gray value is obtained by calculating the power of the inverse of the *Midtone* with the ratio of *GrayDiff* to *Diff* as the base and multiplying by 255, as shown in Equations (17–20).


(17) Diff=threHigh-threShadow

(18) GrayDiff=Gray-threShadow

(19) $$Midtone=Midtone_{0}-M_{4}$$

(20) $$Gray^{\prime }=\left\{ \begin{array}{cc} 0, & GrayDiff\leq 0\\ 255\times {\left(\frac{GrayDiff}{Diff}\right)}^{\frac{1}{Midtone}}, & GrayDiff>0 \end{array}\right.$$

where *Midtone*_0_ is denoted as the initial midtone value and *M*_4_ is the amount of change. After the above adjustment, the grayscale image of the input color scale adjusted by the input color scale *I*_in_(*x*, *y*) is obtained. Then, the ratio coefficients of the deviation of the white field threshold *outHigh* and the black field threshold *outShadow* and 255 in the output color scale are calculated. After a series of calculations, the color-adjusted grayscale image is obtained as the updated mask mapping, which is I_4_(*x*, *y*), as shown in Equation (21).


(21) $$I_{4}\left(x,y\right)=\frac{outHigh-outShadow}{255}\cdot I_{\text{in}}\left(x,y\right)+outShadow$$

In this paper, the value of *threShadow* is 86 and *threHigh* is 255; the value of *outShadow* is 0 and *outHigh* is 255. The dynamic adjustment of the color scale is done by dynamically and linearly adjusting the middle tone value *M*_4_ for real-time rendering to simulate the change process of wheat stripe rust.

#### Scatter symptom simulation

Scatter symptom is characterized by the natural distribution of the spots on the plant surface, mostly scattered, rarely in patches, with a relatively smooth surface. In this paper, we take rose black spot as the specific object of study to realize the simulation of scatter symptom.

**FIGURE 7:**
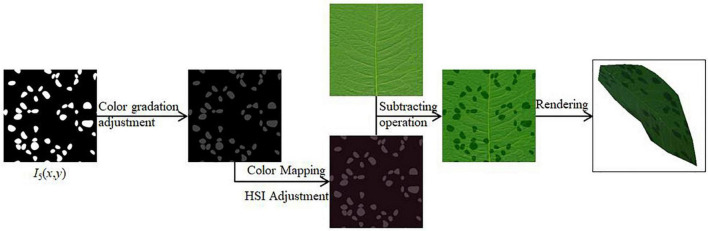
Schematic diagram of rose black spot simulation.

In this paper, we use the Perlin noise function to perturb the regular circular spots in two-dimensional space in terms of distribution and shape, respectively, so that we can generate the disease spots that meet the characteristics of scattered morphological symptoms, as shown in Figure 7. The algorithm process steps are as follows.

(1) The Perlin noise function is used as a random function to generate a number of regular circles for random distribution in the 2D plane, and the dynamic scaling of the radius of the circles can control the size of the spots. After adjustment, the scaling value of Perlin noise used here for position perturbation is set to 32. The larger the scaling value, the more intensive the Perlin noise calculation.
(2) A random function is used to affect the size of the generated regular circles, setting the random range of shape scaling multipliers between 0.5 and 1.0. The Perlin noise function is again used, here scaled to a value of 8, to perturb the regular circular shape to deform it, thus generating an irregular speckle pattern.
(3) The white patches generated above to represent the diseased spots are adjusted in gray scale. After performing color mapping, the color of the disease spots is adjusted by adjusting the value of HSI (Zhi et al., 2020). An image subtraction operation is performed with the original leaf texture mapping to generate the scattered spots of the disease in 2D view. After applying it to the 3D model, the final rendering result is obtained.

The number of scattered spots is predetermined for the background program. According to step (1) above, the scattered spot locations of rose black spot are determined by Perlin noise as a random generator. Each random point generated by the random function corresponds to some random value in the interval.

The random value corresponding to the *i* random point is *value*_*i*_, and the threshold that can be changed in real time for judgment is *M*_5_. *Display*(*i*) is the function that determines whether each random point will be shown to be rendered as a disease spot, as shown in Equation (22).


(22) $$Display\left(i\right)=\left\{ \begin{array}{c} True,value_{i}\leq M_{5}\\ False,value_{i}>M_{5} \end{array}\right.$$

Each random point initially generated is traversed, and when the corresponding random value is less than or equal to the set threshold value indicates that the point is displayed, otherwise, the point is not displayed, thereby updating the current spot texture mapping *I*_5_(*x*, *y*).

### Simulated similarity test

Convolutional neural network is the leading architecture for image classification, recognition, and detection tasks in deep learning (Rawat and Wang, 2017; Li et al., 2020). In this paper, real images are used as the training set and the model is trained using ResNet (He et al., 2016). The simulation results are used as a test set to get their recognition accuracy of disease symptom types as a way to complete the simulation similarity test.

#### Structural design of ResNet model

**FIGURE 8:**
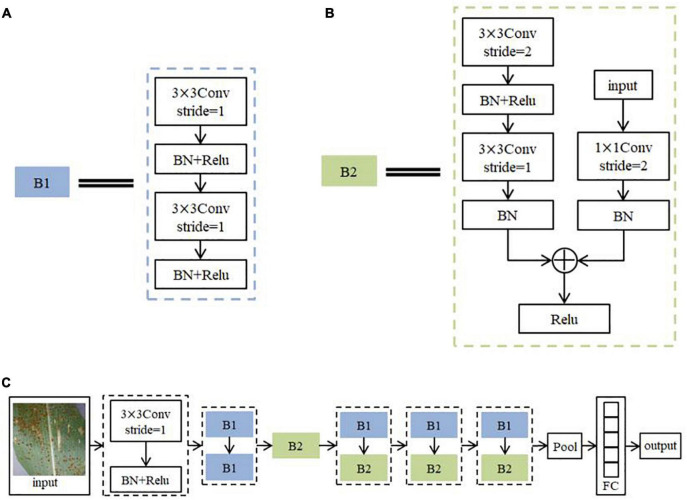
Adjusted ResNet network structure. **(A)** B1, **(B)** B2, **(C)** overall structure.

The advanced nature of the ResNet model allows its structure to be changed and adapted flexibly according to the requirements. The network structure built in this paper is shown in Figure 8. It consists of 56 layers of network. Among them, Conv is the convolutional layer and stride is the step size. BN is Batch Normalization, which aims to regularize the image (Zhu et al., 2017). The activation function is Relu. Pool is the pooling layer. FC is the Fully Connected Layers. Because there are two sequences of steps with repeated operations in the ResNet model for feature extraction of image information, the steps with repeated operations are directly summarized into two different modules B1 and B2 to simplify the structure diagram in order to represent the network structure more clearly. The practical role of both modules is to continuously extract the feature information of the image. The algorithm flow steps of the model are as follows.

(1) Initial feature extraction is performed on the training set images using a convolution kernel of size 3 × 3 with a step size of 1. The BN layer is used to normalize the disease features. ReLU activation function (Lin and Shen, 2018) is used to non-linearize the disease features. Each subsequent convolutional calculation is followed by batch normalization and activation function, which will not be reviewed later.
(2) The image features are further extracted and fused with the input feature information using 2 sets of convolution kernels of size 3 × 3 with a step size of 1. This process is seen as the overall module B1 (Figure 8A), and the B1 calculation operation is repeated twice.
(3) Deep features are extracted using one set of convolutional kernels of size 3 × 3 with a step size of 2 and one set of convolutional kernels of size 3 × 3 with a step size of 1. The result of this step is added to the result obtained by computing the original input image using a set of convolutional kernels of size 1 × 1 with step size 2, and the result after the summation is converted to a function using the activation function. This process may be seen as overall module B2 (Figure 8B).
(4) The calculation process of B1 and B2 is repeated three times in order to further extract the deep features of the image.
(5) When the network finishes processing the image with feature extraction, the feature map is compressed using the average pooling operation to reduce the amount of network computation. Finally, Softmax classifier (Zeng et al., 2014) is used to output the probabilities of the corresponding categories through a fully connected layer of size 5. The label with the highest probability is output as the predicted classification result.

#### Data acquisition and pre-processing

The experimental images collected for training in this paper consist of PlantVilage (Hughes and Salathe, 2015), a publicly available online dataset for plant disease image classification, and a self-built dataset. A total of 1,000 RGB color images of different disease symptom types are collected. The experimental images collected for testing are all obtained simulated result maps, with a total of 200 RGB color images. The self-built dataset was obtained from the image data crawled in the Agroforestry Science website using crawler software, and the useless data were removed by manual screening. Under the guidance and advice of agroforestry-related professionals, we finished organizing the image data and tagging the category labels. In this paper, the pixel size of all images was adjusted to 256 × 256 × 3, and the original images with less than 256 × 256 × 3 pixels were zero-filled. In order to obtain experimental images that better meet the training requirements, some of the images in this paper are adjusted in terms of sharpness, contrast, sharpening, and interference information processing.

#### Result of similarity test

The parameters of the model training were set as follows: the learning rate was set to 0.005, the number of iterations was 600 rounds, and the loss function was the cross-entropy loss function, and the training accuracy could reach 98.1%. The performance of the model is evaluated by randomly taking 20% of the real image dataset as the validation set, and the accuracy obtained is 92%, which is a high recognition accuracy, indicating that the model can be used to simulate the similarity test.

The simulated result maps of each type of plant diseases were identified as a test set, so as to achieve the purpose of similarity test proposed in this paper, and the overall accuracy of the test obtained is 87%. Because there are certain differences between simulated results and real images, some interference factors are difficult to avoid, including the difference between 3D models and real plants, the difference between the apparent texture of simulated diseases and real diseases, and the color space ratio, etc., the recognition accuracy will be significantly lower than that of the validation set when the test set is simulated results. The formula for calculating the recognition accuracy of different symptom types is shown in Equation (23):


(23) $$Ob_{\text{k}}=\frac{Correct_{\text{k}}}{Correct_{\text{k}}+Error_{\text{k}}}\times 100\%$$

where *Ob*_k_ refers to the recognition accuracy of the *k* symptom, *Correct*_k_ refers to the number of image samples with correct recognition results for the *k* symptom type, and *Error* refers to the number of image samples with incorrect recognition results for the *k* symptom.

The obtained recognition for each type is shown in the Table 1. It can be seen that the overall results of the simulated similarity test using deep learning are good. Ring pattern has the most distinctive features and is significantly different from other symptom types, with the highest recognition accuracy. In contrast, the plant diseases of rust spot are more easily misidentified as powdery mildew or scatter types. The formation of rust spot at a certain period of time is similar in distribution and shape to these two symptom types, and the identification accuracy is relatively lowest.

**TABLE 1:** Recognition accuracy of different disease symptom types.

Symptom	Number of test images	Number of misclassifications	Recognition accuracy
Discoloration	40	5	87.5%
Powdery mildew	40	3	92.5%
Ring pattern	40	2	95.0%
Rust spot	40	11	72.5%
Scatter	40	5	87.5%

## Results

### Display interface operation

In this paper, we analyze the functional requirements of the plant disease simulation user interface and design a simulation display interface based on a message-driven model instead of a command-line program using Unity. Users can: (1) select the simulation object and open the model file (.obj file), set the growth conditions of temperature and humidity, and enter the simulation algorithm process of the corresponding object; (2) slide the time module to observe the change process, and the system writes the current rendering time and real-time frame rate to the real-time information area in real time; (3) use the right mouse button to rotate the model. The W, S, A, and D keys of the keyboard control the zoom in, zoom out, left, and right movement of the model, respectively. The W, S, A, and D keys of the keyboard can control the zoom in, zoom out, left and right movement of the model, respectively.

**FIGURE 9:**
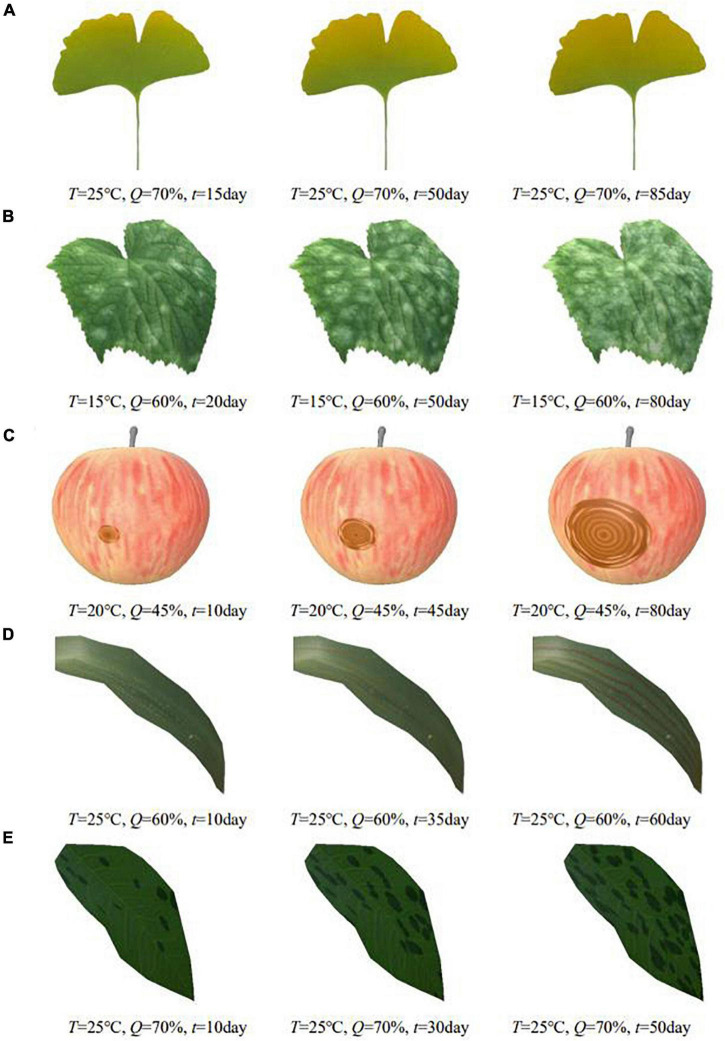
Simulation results of different plant diseases under different parameters control. **(A)** Ginkgo yellows, **(B)** cucumber powdery mildew, **(C)** apple ring rot, **(D)** wheat stripe rust, **(E)** rose black spot.

### Experimental results

In order to show the simulation effect clearly and intuitively, the complex plant model is pre-processed in this paper, and only the parts of plant organs with diseases are reserved for display. The average frame rates of different plant disease simulations are shown in Table 2, indicating that the simulation can be performed efficiently in real time. Figure 9 shows the simulation results of the above plant diseases under different environmental conditions and different disease occurrence times. It can be seen that the severity of plant disease damage to the plant epidermis increases with time, gradually spreading to infest the entire surface of the organ when the temperature and humidity are in the right range for the growth of the disease.

**TABLE 2:** Average frame rates of different plant diseases.

Symptom	Plant disease	Simulation results	Average frame rate
Discoloration	Ginkgo yellows	Figure 9A	361
Powdery mildew	Cucumber powdery mildew	Figure 9B	330
Ring pattern	Apple ring rot	Figure 9C	324
Rust spot	Wheat stripe rust	Figure 9D	293
Scatter	Rose black spot	Figure 9E	338

## Discussion

**FIGURE 10:**
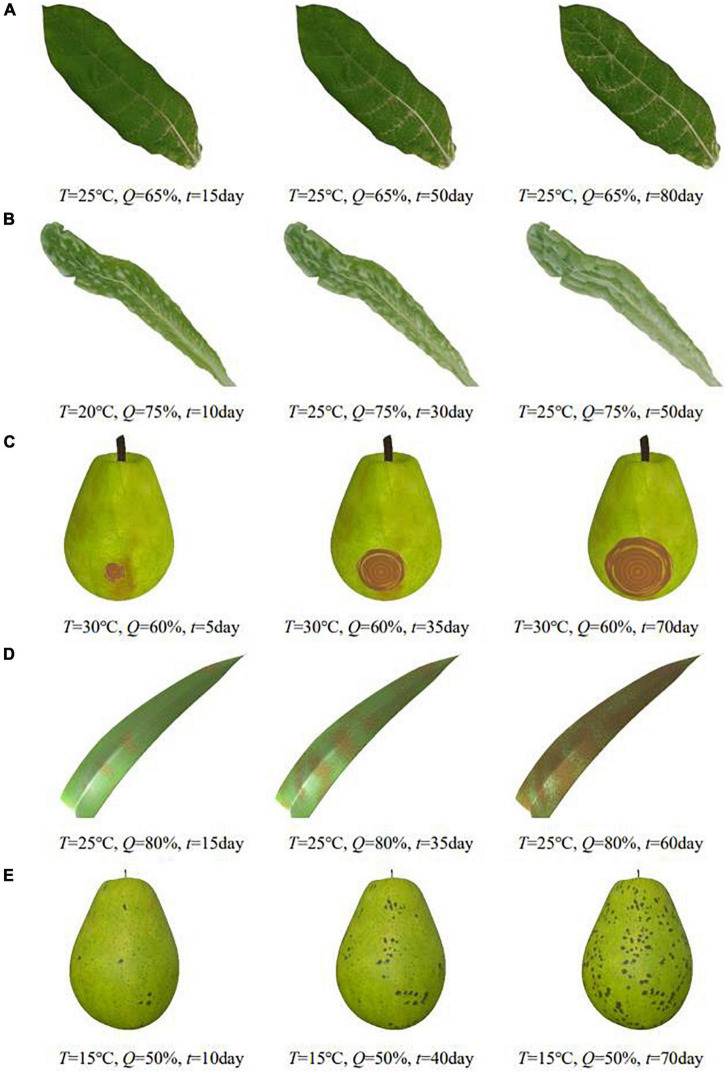
Simulation results of other plant diseases under different parameters control. **(A)** Tobacco mosaic, **(B)** lettuce downy mildew, **(C)** pear ring rot, **(D)** corn rust, **(E)** pear scab.

Plant diseases are diverse and complex. The number of phenological patterns under the influence of different exogenous and endogenous conditions is even more uncountable. When visualizing them, it is difficult to classify and simulate plant diseases from a plant pathology perspective. In this regard, this paper defines a common relationship between the diffusion process of plant diseases under the influence of environment and designs a generic time-varying model of plant disease. In this paper, by observing and analyzing the apparent symptoms of diseases, we classify plant diseases by symptom characteristics and propose an apparent simulation algorithm to realize visual simulation of different plant diseases. To verify the generalizability of the algorithm, this paper implements the apparent simulation of five other plant diseases using the proposed five symptom simulation algorithms, respectively, as shown in Figure 10.

In addition to the proposed deep learning-based similarity check method, to be able to evaluate the simulation results more comprehensively, this paper designs the “Questionnaire on the Effectiveness of Plant Disease Simulation Based on Feature Classification.” We invite users to visually compare the simulation results with real pictures. Using a Likert scale, users rated the simulation results quantitatively and made suggestions for optimization, and the questionnaire data were analyzed using SPSS software (Pallant, 2013). In order to be able to cover different types of users to participate in the evaluation, users of different age groups, different educational stages and different industries were invited to this paper, and a total of 242 valid questionnaires were collected. The age groups covered from below 16 to above 45 years old, with the age group of 16–35 years old dominating; the education levels covered from junior high school to above master’s degree, with bachelor and master’s degree dominating; the professions included agriculture and forestry related, computer related and other professions, with reasonable composition. Descriptive analysis of the overall effect evaluation was conducted, and the results obtained are shown in Table 3. It can be obtained that the median evaluation score of each symptom type is 4, which indicates that it is similar, indicating that the overall simulation effect meets expectations and is recognized by users.

**TABLE 3:** Descriptive statistics.

Symptom	Minimum value	Maximum value	Median	Average value	Standard deviation	Variance
Discoloration	1	5	4	3.909	0.815	0.664
Powdery mildew	1	5	4	3.843	0.835	0.697
Ring pattern	1	5	4	4.033	0.799	0.638
Rust spot	1	5	4	3.760	0.860	0.739
Scatter	1	5	4	4.041	0.814	0.662

Combined with the shortcomings and suggestions made by users on the simulation results collected from the non-scale questions in the questionnaire, they are summarized as follows: in terms of details, users suggested that the gradient texture of the simulated area of discoloration is not obvious, the stacking effect of powdery mildew needs to be refined, the boundary treatment of ring pattern is not detailed enough, the color of rust spot needs to be further processed, and the apparent differentiation between different periods of scatter is not enough. These also provide valuable reference directions for the subsequent optimization work.

## Conclusion

The time-varying generic model proposed in this paper simplifies the unqualified and complex processes into quantitative common relationships in a uniform computational manner. It can also set different influence coefficients to express the variability of plant diseases by the action of influencing factors, effectively integrating algorithmic resources. The simulation algorithm proposed in this paper for different disease symptoms generates the texture of disease spots in two-dimensional space, and then renders them on the three-dimensional model to get the final effect. For discoloration, this paper mainly uses the three-stage gray-scale remapping to realize the discoloration simulation with a sense of hierarchy; for powdery mildew, this paper combines Worley noise and Perlin noise application to realize the simulation; for ring pattern, this paper combines image processing and noise disturbance deformation to simulate the pattern of spots into two parts: initial water-stained spots and concentric circles; for rust spot, this paper uses mask mapping to mark specific onset areas, simulates the raised particles of rust spots through bump mapping, and uses color scale adjustment to complete the changes of spot texture; for scatter, this paper makes double application of Perlin noise to represent the distribution of spots and disturbance rule shape, and sets dynamic thresholds to complete the simulation of scatter from less to more. In the simulation similarity test, the recognition accuracy reached 87%, indicating that the disease phenology simulation algorithm in this paper can effectively and realistically realize the process simulation of different plant diseases. The overall complexity of the algorithm is moderate, and it operates efficiently, which provides a new solution for disease simulation research and can be extended to more types of disease simulation. In the future, we will work on three aspects: enriching the types of disease symptoms, optimizing the general model of disease time variation, and improving the overall functions to increase the freedom of simulation.

## Data availability statement

The original contributions presented in this study are included in the article/supplementary material, further inquiries can be directed to the corresponding author.

## Author contributions

Both the authors made substantial, direct, and intellectual contributions to this work, discussed and revised the manuscript, and approved the submitted version.

## Conflict of interest

The authors declare that the research was conducted in the absence of any commercial or financial relationships that could be construed as a potential conflict of interest.

## Publisher’s note

All claims expressed in this article are solely those of the authors and do not necessarily represent those of their affiliated organizations, or those of the publisher, the editors and the reviewers. Any product that may be evaluated in this article, or claim that may be made by its manufacturer, is not guaranteed or endorsed by the publisher.

## References

[B1] Alsweis M. Deussenn O. Liu J. (2017). Simulation and visualization of adapting venation patterns. *Comput. Animat. Virtual Worlds* 28 1–11. 10.1002/cav.1723

[B2] Bellini R. Kleiman Y. Cohen-Or D. (2016). Time-varying weathering in texture space. *ACM Trans. Graph.* 35 1–11. 10.1145/2897824.2925891

[B3] Cavalier A. Gilet G. Ghazanfarpour D. (2019). Local spot noise for procedural surface details synthesis. *Comput. Graph.* 85 92–99. 10.1016/j.cag.2019.10.003

[B4] Chappell T. M. Codod C. B. Williams B. W. Kemerait R. C. Culbreath A. K. Kennedy G. G. (2020). Adding epidemiologically important meteorological data to peanut Rx, the risk assessment framework for spotted wilt of peanut. *Phytopathology* 110 1199–1207. 10.1094/PHYTO-11-19-0438-R 32133919

[B5] Chen G. Chen B. Liu Y. Li H. (2018). Research on complex 3D tree modeling based on l-system. *IOP Conf.* 322:062005. 10.1088/1757-899X/322/6/062005

[B6] Chen L. Yuan Y. Song S. (2017). “Hierarchical denoising method of crop 3D point cloud based on multiview image reconstruction,” in *Proceedings of The International Conference on Intelligent Agriculture 2017*, eds Li D. Zhao C. (Cham: Springer), 431–442. 10.1007/978-3-030-06137-1_38

[B7] Dickinson M. (2020). Plant pathology and plant diseases. *Plant Pathol.* 69 1812–1812. 10.1111/ppa.13280

[B8] Fan D. Liu S. Wei Y. (2013). “Fruit ring rot simulation based on reaction-diffusion model,” in *Proceedings of the International Conference on Virtual Reality & Visualization* (Xi’an: IEEE), 199–205. 10.1109/ICVRV.2013.38

[B9] Guingo G. Sauvage B. Dischler J. M. Cani M. P. (2017). Bi-layer textures: a model for synthesis and deformation of composite textures. *Comput. Graph. Forum* 36 111–122. 10.1111/cgf.13229

[B10] He K. Zhang X. Ren S. Sun J. (2016). “Deep residual learning for image recognition,” in *Proceedings of the 2016 IEEE Conference on Computer Vision and Pattern Recognition*, Las Vegas, NV, 770–778. 10.1109/CVPR.2016.90

[B11] Hughes D. P. Salathe M. (2015). An open access repository of images on plant health to enable the 580 development of mobile disease diagnostics. *arXiv* [Preprint].

[B12] Ishitobi A. Nakayama M. Fujishiro I. (2020). Visual simulation of weathering coated metallic objects. *Vis. Comput.* 36 2383–2393. 10.1007/s00371-020-01947-w

[B13] Isokane T. Okura F. Ide A. Matsushita Y. Yagi Y. (2018). “Probabilistic plant modeling via multi-view image-to-image translation,” in *Proceedings of the 2018 IEEE/CVF Conference on Computer Vision and Pattern Recognition (CVPR)* (Salt Lake City, UT: IEEE), 2906–2915. 10.1109/CVPR.2018.00307

[B14] Jeong S. H. Park S. H. Kim C. H. (2013). Simulation of morphology changes in drying leaves. *Comput. Graph. Forum* 32 204–215. 10.1111/cgf.12009

[B15] Kamata Y. Manabe Y. Yata N. (2014). “Simulation of aging metal with preservative coating,” in *Proceedings of the 2013 International Conference on Computer Graphics, Visualization, Computer Vision, and Game Technology* (Amsterdam: Atlantis Press), 46–51. 10.2991/visio-13.2014.8

[B16] Kider J. T. Raja S. Badler N. I. (2011). Fruit senescence and decay simulation. *Comput. Graph. Forum* 30 257–266. 10.1111/j.1467-8659.2011.01857.x

[B17] Li D. Deng L. Cai Z. (2020). Research on image classification method based on convolutional neural network. *Neural Comput. Appl.* 33 8157–8167. 10.1007/s00521-020-04930-7

[B18] Lin G. Shen W. (2018). Research on convolutional neural network based on improved ReLU piecewise activation function. *Procedia Comput. Sci.* 131 977–984. 10.1016/j.procs.2018.04.239

[B19] Liu S. Fan D. (2015). Computer modeling and simulation of fruit sunscald. *Int. J. Image Graph.* 15 1550013.1–1550013.18. 10.1142/S0219467815500138

[B20] Liu Z. Shen C. Li Z. Weng T. Deussen O. Cheng Z. (2019). “Interactive modeling of trees using VR devices,” in *Proceedings of the 2019 International Conference on Virtual Reality and Visualization (ICVRV)*, Hong Kong, 69–75. 10.1109/ICVRV47840.2019.00020

[B21] Liu Z. Wu K. Guo J. Wang Y. Deussen O. Cheng Z. (2021). Single image tree reconstruction via adversarial network. *Graph. Models* 2021:101115. 10.1016/j.gmod.2021.101115

[B22] Miao T. Zhao C. Guo X. Lu S. Wen W. (2014). Visual simulating appearance of plant leaves infected by disease and insect pests. *Trans. Chin. Soc. Agric. Eng.* 30 169–175. 10.3969/j.issn.1002-6819.2014.02.022

[B23] Moyer M. M. Gadoury D. M. Wilcox W. F. Seem R. C. (2016). Weather during critical epidemiological periods and subsequent severity of powdery mildew on grape berries. *Plant Dis.* 100 116–124. 10.1094/PDIS-12-14-1278-RE 30688564

[B24] Munoz-Pandiella I. Bosch C. Merillou N. Patow G. Merillou S. Pueyo X. (2018). Urban weathering: interactive rendering of polluted cities. *IEEE Trans. Vis. Comput. Graph.* 2018 1–14. 10.1109/TVCG.2018.2794526 29994511

[B25] Pallant J. (2013). SPSS survival manual: a step by step guide to data analysis using SPSS for windows. *Austral. N. Zealand J. Public Health* 37 597–598. 10.1111/1753-6405.12166

[B26] Perlin K. (1985). An image synthesizer. *ACM Siggraph Comput. Graph.* 19 287–296. 10.1145/325165.325247

[B27] Pieruschka R. Schurr U. (2019). Plant phenotyping: past, present, and future. *Plant Phenomics* 2019 179–184. 10.1155/2019/7507131 PMC7718630 33313536

[B28] Rawat W. Wang Z. (2017). Deep convolutional neural networks for image classification: a comprehensive review. *Neural Comput.* 29 2352–2449. 10.1162/NECO_a_00990 28599112

[B29] Scholthof K. B. G. (2007). The disease triangle: pathogens, the environment and society. *Nat. Rev. Microbiol.* 5 152–156. 10.1038/nrmicro1596 17191075

[B30] Shang S. Shang Z. Yu J. Zhang X. Wu J. Jiang D. (2018). Impacts of climate change on *Fusarium* head blight in winter wheat. *Fresenius Environ. Bull.* 27 3906–3913.

[B31] Tan W. (1991). A generalized model for simulating the temporal dynamics of plant disease epidemics – Richards function. *Acta Phytopathol. Sin.* 21 235–240. 10.13926/j.cnki.apps.1991.03.026

[B32] Tang Y. Wu D. Y. Fan J. (2013). Computational approach to seasonal changes of living leaves. *Comput. Math. Methods Med.* 2013:619385. 10.1155/2013/619385 23533545 PMC3596921

[B33] Wen W. Wang Y. Wu S. Liu K. Gu S. Guo X. (2021). 3D phytomer-based geometric modelling method for plants—the case of maize. *AoB Plants* 13 1079–1087. 10.1093/aobpla/plab055 34603653 PMC8482417

[B34] Worley S. (1996). “A cellular texture basis function,” in *Proceedings of the Conference on Computer Graphics & Interactive Techniques* (Trier: DBLP), 291–294. 10.1145/237170.237267

[B35] Wu S. Xiao B. Miao T. Wen W. Guo X. (2018). An interactive design method for realistic fruit rot modeling and simulation. *Int. J. Model. Simul. Sci. Comput.* 9 1850038.1–1850038.14. 10.1142/S1793962318500381

[B36] Xu J. Miao T. Yang T. Xu T. Ji J. Jin L. (2017). Method for three dimensional visualization of plant lesion appearance. *Bangl. J. Bot.* 46 1079–1087.

[B37] Zeng R. Wu J. Shao Z. Senhadji L. Shu H. (2014). Quaternion softmax classifier. *Electron. Lett.* 50 1929–1931. 10.1049/el.2014.2526 35526269

[B38] Zhang F. Liu Z. Cheng Z. Deussen O. Chen B. Wang Y. (2021). “Mid-air finger sketching for tree modeling,” in *Proceedings of the 2021 IEEE Virtual Reality and 3D User Interfaces (VR)* (Lisboa: IEEE). 10.1109/VR50410.2021.00110

[B39] Zhang J. Duan F. Zhou M. Jiang D. Wang X. Wu Z. (2018). Stable and realistic crack pattern generation using a cracking node method. *Front. Comput. Sci.* 12 777–797. 10.1007/s11704-016-5511-9

[B40] Zhang Y. Geng G. Zhou M. (2014). The simulation of rusty phenomenon based on image texture feature. *Appl. Mech. Mater.* 513–517 3972–3975.

[B41] Zhi S. Cui Y. Deng J. Du W. (2020). An FPGA-based simple RGB-HSI space conversion algorithm for hardware image processing. *IEEE Access* 8 173838–173853. 10.1109/ACCESS.2020.3026189

[B42] Zhu W. Qu J. Wu R. (2017). Straight convolutional neural networks algorithm based on batch normalization for image classification. *J. Comput. Aided Design Comput. Graph.* 29 1650–1657.

